# Characterization of silicon heterojunctions for solar cells

**DOI:** 10.1186/1556-276X-6-152

**Published:** 2011-02-16

**Authors:** Jean-Paul Kleider, Jose Alvarez, Alexander Vitalievitch Ankudinov, Alexander Sergeevitch Gudovskikh, Ekaterina Vladimirovna Gushchina, Martin Labrune, Olga Alexandrovna Maslova, Wilfried Favre, Marie-Estelle Gueunier-Farret, Pere Roca i Cabarrocas, Eugene Ivanovitch Terukov

**Affiliations:** 1 Laboratoire de Génie Electrique de Paris, CNRS UMR 8507, SUPELEC, Univ P-Sud, UPMC Univ Paris 6, 11 rue Joliot-Curie, Plateau de Moulon, 91192 Gif-sur-Yvette Cedex, France; 2 A.F. Ioffe Physico-Technical Institute, Polytechnicheskaya Str. 26, St. Petersburg, 194021, Russia; 3 St. Petersburg Academic University-Nanotechnology Research and Education Centre RAS, Hlopina Str. 8/3, St. Petersburg, 194021, Russia; 4 Laboratoire de Physique des Interfaces et des Couches Minces, Ecole Polytechnique, CNRS, 91128 Palaiseau, France; 5 TOTAL S.A., Gas & Power-R&D Division, Courbevoie, France

## Abstract

Conductive-probe atomic force microscopy (CP-AFM) measurements reveal the existence of a conductive channel at the interface between *p*-type hydrogenated amorphous silicon (*a*-Si:H) and *n*-type crystalline silicon (*c*-Si) as well as at the interface between *n*-type *a*-Si:H and *p*-type *c*-Si. This is in good agreement with planar conductance measurements that show a large interface conductance. It is demonstrated that these features are related to the existence of a strong inversion layer of holes at the *c*-Si surface of (*p*) *a*-Si:H/(*n*) *c*-Si structures, and to a strong inversion layer of electrons at the *c*-Si surface of (*n*) *a*-Si:H/(*p*) *c*-Si heterojunctions. These are intimately related to the band offsets, which allows us to determine these parameters with good precision.

## Introduction

In the field of silicon solar cells, recent progress has been achieved in two directions: silicon heterojunctions and silicon nanowires. These two topics are briefly addressed here and we show some new characterization results that use conductive-probe atomic force microscopy (CP-AFM) measurements.

Silicon heterojunctions are formed between crystalline silicon (*c*-Si) and hydrogenated amorphous silicon (*a*-Si:H). Solar cell efficiencies of up to 23% have been demonstrated on high quality *n*-type *c*-Si wafers with layers of *p*-type *a*-Si:H deposited at the front (as the emitter) and *n*-type *a*-Si:H deposited at the back (as the back surface field), respectively [1]. Since transport properties are quite poor in *a*-Si:H due to the large amount of defects and band gap states and low carrier mobilities, the doped *a*-Si:H layers are used to form the junctions, but their thickness has to be kept very low. The front *a*-Si:H layer has to be very thin in order to minimize absorption of incoming photons and to privilege absorption in *c*-Si. One key feature of the Si heterojunctions is the very good passivation property of the *c*-Si surface by *a*-Si:H. This is even improved by inserting a thin undoped *a*-Si:H layer (so-called "intrinsic" layer, which leads to the "HIT"-heterojunction with intrinsic thin layer denomination [2]). This limits interface recombination and leads to very high open circuit voltages [3]. Band offsets between *a*-Si:H and *c*-Si also play a crucial role because they determine the band bending, which influences the carrier collection. We here demonstrate the existence of a conduction channel along both the (*n*) *a*-Si:H/(*p*) *c*-Si and the (*p*) *a*-Si:H/(*n*) *c*-Si interfaces from direct CP-AFM measurements performed on cleaved sections of solar cells. We show from additional planar conductance measurements and simulations that these are related to strong inversion regions at the interfaces. From the temperature dependence, we determine the values of band offsets.

## Experimental details

### Solar cell structure

**Figure 1:**
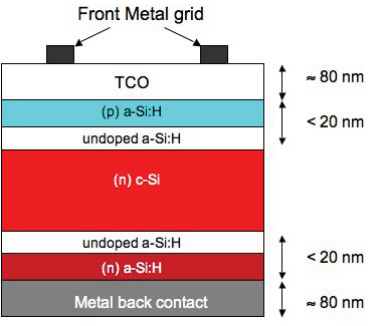
**Cross-section of a silicon heterojunction solar cell on *n*-type *c*-Si**.

A typical solar cell structure based on *a*-Si:H/*c*-Si heterojunctions formed with *n*-type *c*-Si is presented in Figure 1. A similar structure stands for *p*-type *c*-Si, replacing the *n*-type *a*-Si:H by *p*-type *a*-Si:H and vice versa. For *n*-type *c*-Si, we used Float Zone, *n*-type *c*-Si wafers, 〈100〉 oriented, with resistivity: *ρ *= 1-5 Ω cm, and thickness: *W *= 300 μm. For the *p*-type *c*-Si, we used Czochralski (CZ) *c*-Si wafers, 〈100〉 oriented, with resistivity: *ρ *= 14-22 Ω cm, and thickness: *W *= 300 μm. We used indium tin oxide (ITO) as the front transparent conductive oxide (TCO), and aluminum as the back metal contact. The *a*-Si:H layers were deposited at Ecole Polytechnique in a radio frequency (13.56 MHz) plasma-enhanced chemical vapor deposition (PECVD) reactor at a substrate temperature of 200°C. Spectroscopic ellipsometry measurements and modeling were used to check that the deposited silicon thin layers were truly amorphous, and that no epitaxial growth occurred on the *c*-Si substrate.

### CP-AFM

**Figure 2:**
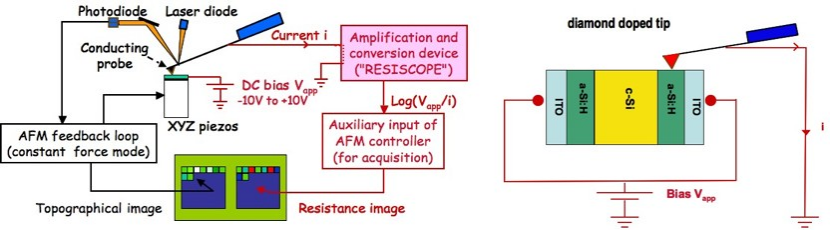
**Sketch of the CP-AFM measurements; left: setup at LGEP with the resiscope extension; right: detail of the sample configuration and biasing**.

CP-AFM measurements were carried out using two different setups (i) in Ioffe Physical-technical Institute (NT-MDT Ntegra Aura) and (ii) in Laboratoire de Génie Électrique de Paris (Digital Instruments Nanoscope IIIa Multimode AFM with the RESISCOPE extension [4]). These setups allow one to apply a stable DC bias voltage to the device and to measure the resulting current flowing through the tip as the sample surface is scanned in contact mode. Schematic AFM setup is shown in Figure 2. In both measurements diamond-coated conductive probes made of silicon were used, the contact interaction force being in the range 100-500 nN. With the help of this technique one can simultaneously examine on the sample cleavages the surface topography and conductive properties of the layers constituting the solar cells. Note that, due to different softwares, the first setup provides images with current values (current flowing through the tip), while the second one provides resistance values, the resistance being defined as the ratio of the applied voltage to the measured current.

**Figure 3:**
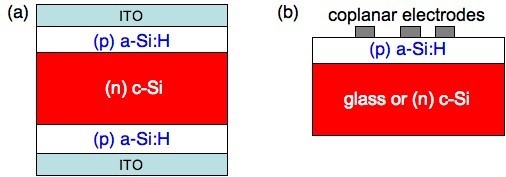
**Sketch of the samples prepared for (a) CP-AFM measurements and (b) planar conductance measurements**.

For these CP-AFM measurements, the normal solar cell structure was replaced by a simpler symmetric configuration, see Figure 3a, where the same *a*-Si:H layer was deposited on both sides of the *c*-Si wafer. Then ITO electrodes were deposited on top of both sides of the wafer, before the sample was cleaved. Some tests were also performed with aluminum instead of ITO as electrodes. The obtained CP-AFM results were globally the same. However, aluminum electrodes formed high ridges at the cleaved edge and their cross-section were poorly conductive due to strong oxidation of aluminum, what induced some problems in AFM imaging. Therefore, here we focus on samples with ITO on both sides. Thus, cleaved sections of ITO/(*n*) *a*-Si:H/(*p*) *c*-Si/ITO and ITO/(*p*) *a*-Si:H/(*n*) *c*-Si/ITO samples with different thicknesses of the *a*-Si:H layer (20, 100, 300 nm) were investigated.

### Planar conductance

The sample structure for these measurements is shown in Figure 3b for *p*-doped *a*-Si:H. The *a*-Si:H layer was deposited in the same run on both *n*-type *c*-Si and glass (Corning 1737). Top coplanar aluminum electrodes were then deposited on the top of *a*-Si:H. We measured the DC current, *I*, resulting from application of a DC bias, *V*, between two adjacent electrodes. We had several electrode designs with various gap distances between them. We checked that the current scaled with the inter-electrode gap distance. We also checked that the current was linearly dependent on the DC voltage, so that we defined the conductance *G *= *I*/*V*. This was then measured as a function of temperature between 150 and 300 K in a cryostat chamber pumped down to 10^-^^5 ^mbar.

The same kind of measurements were also performed on series of samples with *n*-doped *a*-Si:H deposited onto *p*-type *c*-Si and glass.

## Results and discussion

**Figure 4:**
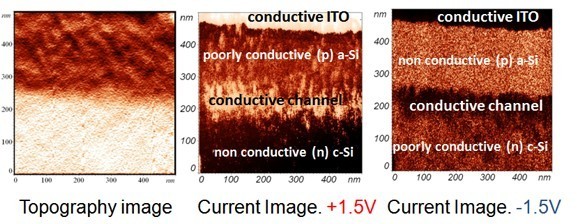
**AFM pictures taken on a cleaved section of an ITO/(*p*) *a*-Si:H/(*n*) *c*-Si/ITO sample**. Left: topography; middle: current image taken at an applied bias of +1.5 V. Right: current image taken at an applied bias of -1.5 V. Typical roughness was less than 5 nm. On the topographical image, the change in height from the dark top region to the light bottom region was of the order of 2 nm. In the current images, the current values ranged from 60 pA to 17 nA.

In Figure 4a,b,c, an example of topography and current images for two different biases, is presented for a (*p*) *a*-Si:H/(*n*) *c*-Si junction. At positive bias applied to the sample, conductive regions appear light in the current images, while for negative bias they appear dark. The current images clearly reveal a conductive interface layer between the *c*-Si substrate and the *a*-Si:H film. This layer is more conductive than both the *c*-Si and *a*-Si:H regions. This conductive interface layer was well observed on all samples for both (*p*) *a*-Si:H/(*n*) *c*-Si and (*n*) *a*-Si:H/(*p*) *c*-Si heterointerfaces whatever the *a*-Si:H layer thickness is. It is worth to note that the conductive layer is not an artifact that could come from the surface roughness. It can be clearly seen when current images are compared with the topography one. There exists one distinct boundary between the *a*-Si:H layer and *c*-Si wafer, and the detected conductive channel lies within *c*-Si substrate.

However, the quantitative results of the interface layer conductivity deduced from CP-AFM measurements have to be considered carefully. Indeed, the reliability of the latter is affected by the quality and nature of the contact between the conductive tip and the sample surface. The sample surface roughness, the AFM tip radius, shape and pressure are well-known factors driving local electrical measurements. Moreover, surface states can induce additional band bending at the tip-surface junction modifying significantly the conductance values [5]. The CP-AFM scanning measurements can also be influenced by the oxidation process after cleaving the sample and the presence of a water meniscus between the tip and the surface that can also lead to tip-induced oxidation or trapping of carriers in localized states [6,7]. The contact between the tip and the cleaved surface can behave as a metal-oxide interface that then determines the current flowing through the tip.

**Figure 5:**
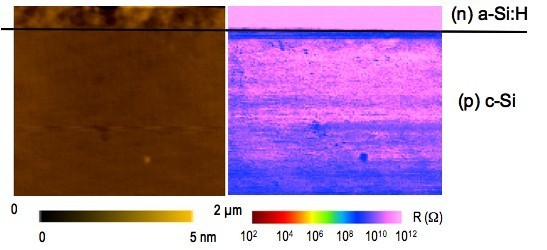
**Topography and electrical image obtained after HF dip at the cleaved section of an (*n*) *a*-Si:H/(*p*) *c*-Si heterojunction**. Left: topography; right: resistance image.

**Figure 6:**
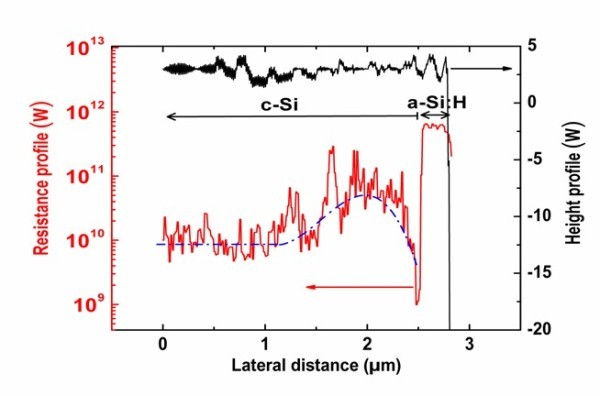
**Profile of local resistance across the (*n*) *a*-Si:H/(*p*) *c*-Si interface corresponding to Figure 5**.

In order to minimize the effects of surface oxide and surface states, CP-AFM measurements were performed at LGEP under nitrogen atmosphere immediately after having dipped the sample in an HF solution. This treatment is known to passivate the silicon surface by reducing the density of silicon dangling bonds, thus minimizing the potential effect of surface states on the surface band bending. Figure 5 illustrates an example of topographical and electrical image of the cleaved section obtained under these conditions with, from top to bottom, the *n*-type *a*-Si:H layer (= 300 nm) and the *p*-type *c*-Si substrate. Contrary to Figure 4, the ITO contact is not observed since it has been partially removed after the HF dip. Compared to results of Figure 4, with the improved measurement procedure, a conductive channel at the (*n*) *a*-Si:H/(*p*) *c*-Si interface is even more clearly observed. The topographic and electrical profiles along the heterointerface presented on Figure 6 show a flat cleaved surface and a higher electrical contrast between the conductive channel and both the *a*-Si:H layer and the *c*-Si substrate. In addition, the electrical image in the *c*-Si also shows a region with increasing conductivity of about 1 μm width when sweeping away from the *a*-Si:H/*c*-Si interface. This can be linked to the depleted space charge region in the low-doped (*p*) *c*-Si (*N*_a _< 10^15 ^cm^-3^), which has a width close to 1 μm.

**Figure 7:**
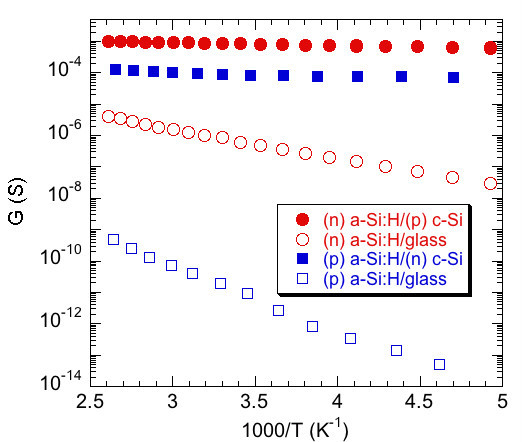
**Arrhenius plots of the planar conductance measured on various samples**. Red circles for (*n*) *a*-Si:H, blue squares for (*p*) *a*-Si:H, full symbols for layers deposited on *c*-Si wafer (on opposite doping type with respect to the deposited *a*-Si:H layer), open symbols for layers deposited on glass.

The existence of an interface conductive channel has also been evidenced by the planar conductance measurements. Indeed, it was shown that the planar conductance was orders of magnitude larger for the samples deposited on *c*-Si substrates (both *n*- and *p*-type) than that measured on the *a*-Si:H layer deposited in the same run on glass substrates. Activation energy of the conductance for the samples deposited on glass was found equal to about 0.35 and 0.2 eV for the (*p*) *a*-Si:H and (*n*) *a*-Si:H layers, respectively [8,9]. These are typical values for doped *a*-Si:H. The conductance for samples deposited on *c*-Si had much lower activation energy, as can be seen in Figure 7. This high planar conductance measured on the samples deposited on *c*-Si is in very good agreement with the presence of the conducting channel revealed by our CP-AFM measurements.

We attribute this thin conductive interface channel along with the low conductance activation energy to a strong inversion layer at the *c*-Si surface that is related to the band offset at the heterojunction.

In order to further demonstrate the existence of the strong interface inversion layer and the related contribution to the conductance, we used the AFORS-HET software [10] to evaluate the free carrier profiles. We introduced the density of states (DOS) typical for *n*-type *a*-Si:H (band gap *E*_g _= 1.75 eV) consisting of two exponential band tails with characteristic energies *k*_B_*T*_C _and *k*_B_*T*_V _of 0.055 and 0.12 eV for the conduction and valence band, respectively, and with a pre-exponential factor of 2 × 10^21 ^cm^-3 ^eV^-1^, and two Gaussian deep defect distributions of donor and acceptor nature being located at 0.58 and 0.78 eV above the top of the valence band, respectively, with a maximum value of 8.7 × 10^19 ^cm^-3 ^eV^-1 ^and a standard deviation of 0.23 eV. A doping density of *N*_d _= 5.34 × 10^19 ^cm^-3 ^was also introduced, setting the Fermi level *E*_F _at 0.2 eV below the conduction band at 300 K, as suggested from the activation energy of the conductance data measured on (*n*) *a*-Si:H samples deposited on glass. The doping density in the crystalline silicon was set at *N*_a _= 7 × 10^14 ^cm^-3^, as found from capacitance versus bias measurements [11], and in agreement with the resistivity of our CZ *c*-Si *p*-type wafers.

**Figure 8:**
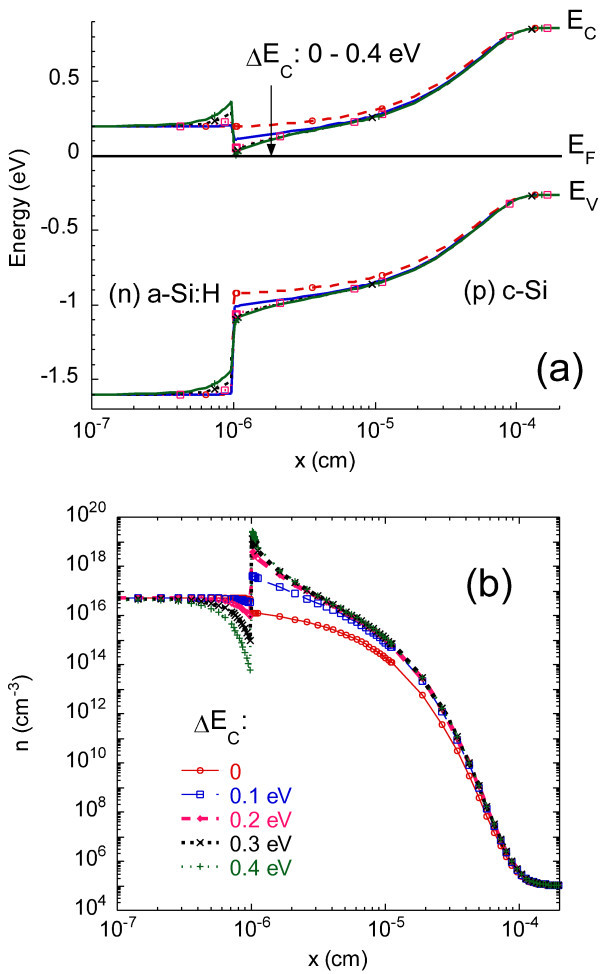
**Modeling of the (*n*) *a*-Si:H/(*p*) *c*-Si heterojunction at equilibrium for various values of the conduction band offset**. **(a) **band diagram, and **(b) **free electron concentration profile.

Figure 8a,b shows the calculated band diagram and the electron concentration profile for various values of the conduction band offset Δ*E*_C _= *E*_C_^*a*^^-Si:H ^- *E*_C_^*c*^^-Si^, respectively. An inversion layer is indeed clearly seen in the interface region of *c*-Si when sticking increase of electron concentration with Δ*E*_C _is observed. On the contrary, increasing Δ*E*_C _leads to a stronger electron depletion in (*n*) *a*-Si:H close to the interface due to a stronger band bending.

**Figure 9:**
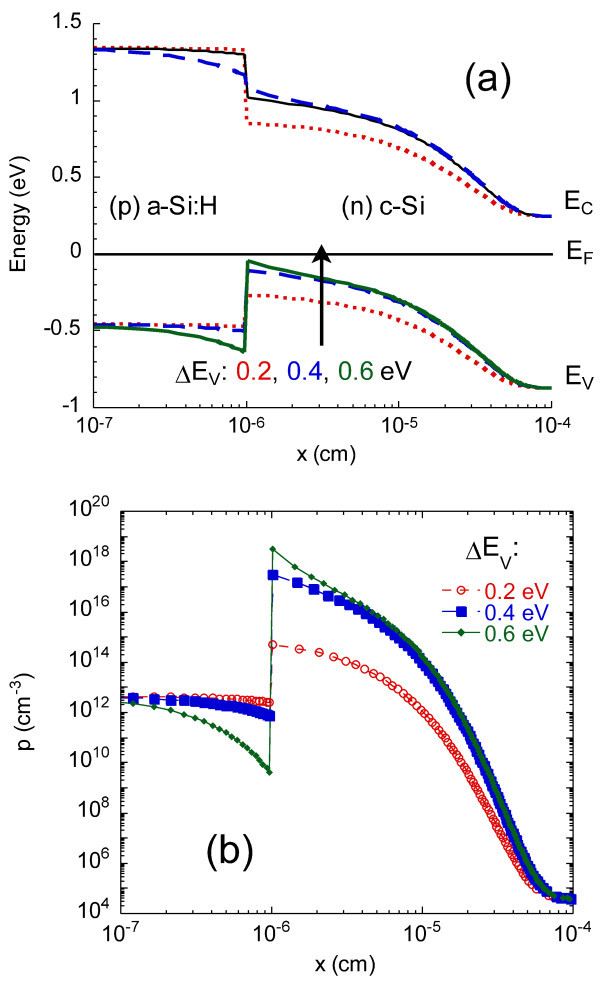
**Modeling of the (*p*) *a*-Si:H/(*n*) *c*-Si heterojunction at equilibrium for various values of the valence band offset**. **(a) **band diagram, and **(b) **free hole concentration profile.

Similar simulations were performed for the (*p*) *a*-Si:H/(*n*) *c*-Si heterojunction. The band gap of *a*-Si:H also was taken at *E*_g _= 1.75 eV, and the position of the Fermi level was fixed at 0.45 eV, which is a reasonable value for *p*-type *a*-Si:H, in agreement with our conductivity measurements. After having introduced the *a*-Si:H parameters, we combined the *a*-Si:H layer with an *n*-type *c*-Si substrate with *N*_d _= 2 × 10^15 ^cm^-3 ^(corresponding to the resistivity value) to simulate the (*p*) *a*-Si:H/(*n*) *c*-Si heterojunction. Calculated band diagram and evaluated hole concentration profiles for different values of valence band offset Δ*E*_V _= *E*_V_^*c*^^-Si ^- *E*_V_^*a*^^-Si:H ^are shown in Figure 9a,b, respectively. Drastic increase of hole concentration is observed in (*n*) *c*-Si layer near the interface for increasing values of band offset, with the appearance of a strong inversion layer for Δ*E*_V _> 0.2 eV. Thus, simulations of both (*n*) *a*-Si:H/(*p*) *c*-Si and (*p*) *a*-Si:H/(*n*) *c*-Si heterojunctions show the appearance of a strong inversion interface region above a given value of band offset. The planar conductance can be related to the carrier density profile. Indeed, the conductance of the strong inversion channel can be written

(1) $$G=\frac{qh}{L}\mu \text{​}N,$$

where *q *is the elementary charge, *h *the length of the coplanar electrodes, *L *the gap between them, *μ *the mobility of the carriers in the strong inversion region, and *N *the sheet carrier density, i.e., the integral over the *c*-Si thickness of the carrier concentration. Carriers to be considered are the electrons for the (*n*) *a*-Si:H/(*p*) *c*-Si interface and the holes for the (*p*) *a*-Si:H/(*n*) *c*-Si interface. We calculated the values of *N *as a function of the band offset and of the temperature. We thus were able to compute the planar conductance and compare it to the experimental data. This proved to be a very precise way to determine the band offsets in the (*n*) *a*-Si:H/(*p*) *c*-Si system [12], where a value of Δ*E*_*C *_= 0.15 eV was found. In the (*p*) *a*-Si:H/(*n*) *c*-Si system, the measured resistance profile was compared to the calculated resistivity profile across the heterojunction. Both profiles have very similar shapes, and the thickness of the strong inversion layer is of the same order of magnitude (50-100 nm). Further analysis of the CP-AFM measurements shows that a strong inversion layer only exists if the valence band offset is large enough, Δ*E*_V _> 0.25 eV [13]. A more detailed theoretical and modeling study including the effect of temperature dependence of the band gaps and of the DOS parameters in *a*-Si:H is under way. It confirms our previous determination of conduction band offset and indicates that the value of valence band offset that best reproduces our experimental data is around Δ*E*_V _= 0.4 eV.

## Conclusion

Silicon heterojunctions were characterized by the CP-AFM technique. A conductive channel between *a*-Si:H layer and *c*-Si substrate was detected in both (*n*) *a*-Si:H/(*p*) *c*-Si and (*p*) *a*-Si:H/(*n*) *c*-Si heterostructures. This conductive channel was attributed to the existence of a strong inversion layer that was also suggested by planar conductance measurements. The existence of this layer can be explained by relatively large band offsets at the heterojunction, as we demonstrated by numerical calculations of the carrier concentration profiles. Comparison with our experimental data allowed us to deduce values of the conduction and valence band offsets.

## Abbreviations

CP-AFM: conductive-probe atomic force microscopy; CZ: Czochralski; DOS: density of states; ITO: indium tin oxide; PECVD: plasma-enhanced chemical vapor deposition; TCO: transparent conductive oxide.

## Competing interests

The authors declare that they have no competing interests.

## Authors' contributions

PRIC and ML deposited the samples. JA, AVA, and EVG carried out CP-AFM measurements. WF carried out planar conductance measurements. ASG and OAM performed modeling. MEGF and EIT participated in the analysis and guidance of the study. JPK supervised the study, participated in the analysis of the results, and drafted the manuscript. All authors read and approved the manuscript.
